# Plantar Molluscum Contagiosum With Dermoscopic Features

**DOI:** 10.5826/dpc.1002a37

**Published:** 2020-04-03

**Authors:** Ömer Faruk Elmas, Asuman Kilitçi

**Affiliations:** 1 Department of Dermatology and Venereology, Faculty of Medicine, Ahi Evran University, Kirsehir, Turkey; 2 Department of Pathology, Faculty of Medicine, Ahi Evran University, Kirsehir, Turkey

**Keywords:** plantar, molluscum contagiosum, dermoscopy, dermatoscopy

## Introduction

Molluscum contagiosum (MC) is a viral infectious skin disease caused by *Molluscum contagiosum* virus. Direct contact with the infected skin and autoinoculation are the main ways of transmission. MC usually presents with pinkish to skin-colored and rounded umbilicated papules varying in size and shape. The diagnosis is usually based on the clinical features [1]. Here we report a case of plantar MC that was initially misdiagnosed as viral wart.

## Case Presentation

**Figure 1:**
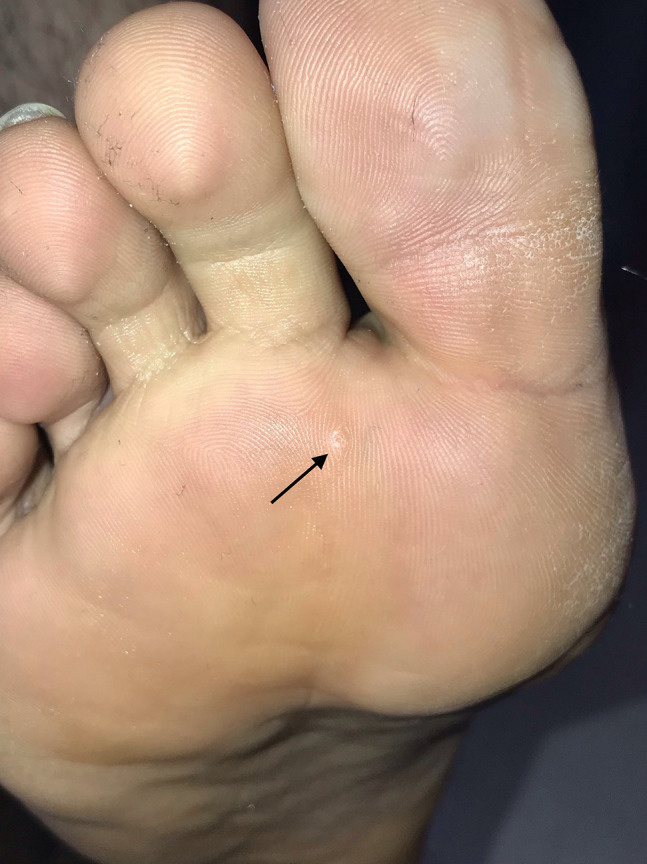
Skin-colored papular lesion on the plantar surface (arrow).

**Figure 2:**
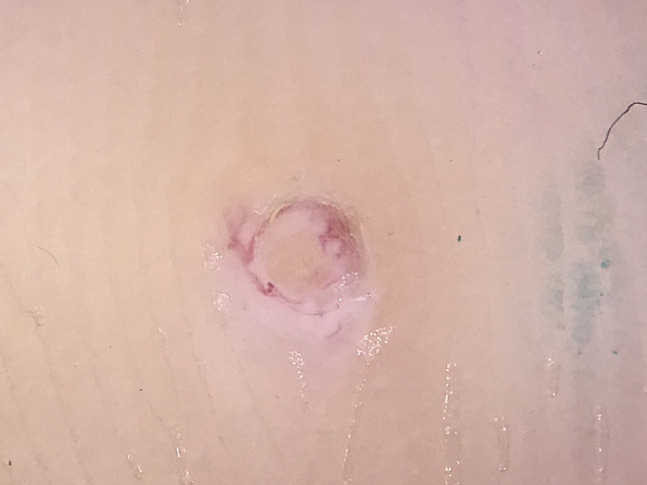
Central yellowish structureless area surrounded by a circumferential thick-branched vessel with bulbous ends.

**Figure 3:**
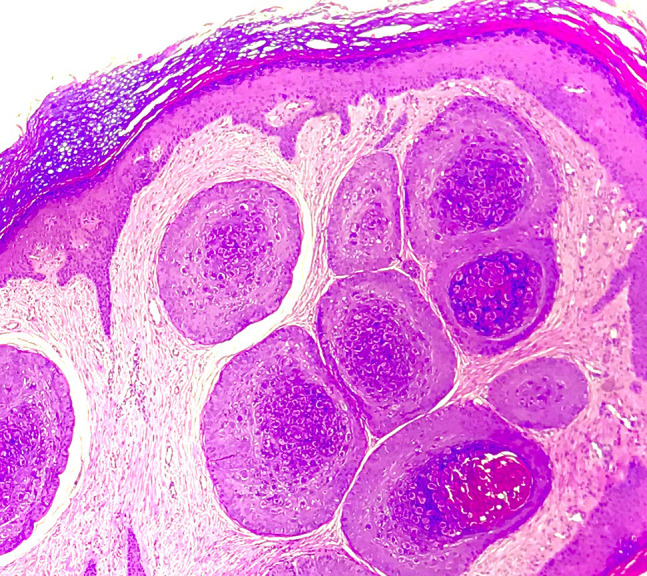
Histopathological examination showed numerous Henderson-Paterson bodies confirming the diagnosis of molluscum contagiosum.

A 28-year-old man having a painless plantar lesion noticed 2 weeks previously was referred to our department with a preliminary diagnosis of plantar wart. Physical examination revealed a 3-mm skin-colored lesion on the plantar surface of the right foot (Figure 1). Dermoscopic examination revealed a central yellowish structureless area surrounded by circumferential thick-branched vessel with bulbous ends (Figure 2). HIV and VDRL (Venereal Disease Research Laboratory) tests were negative. A total shave excision was performed with preliminary diagnoses of MC and viral wart. Histopathological examination showed numerous Henderson-Paterson bodies (intracytoplasmic hyaline eosinophilic inclusion bodies), confirming the diagnosis of MC (Figure 3). The surgical intervention allowed a complete clearance of the lesion and no recurrence was observed after 2 months.

## Conclusions

MC is a common infectious condition that may affect both sexes and all age groups. It may present with single or multiple lesions. Lower abdomen, thighs, genitals, and perianal areas are the common sites involved [1]. Plantar MC is very rare. Subungual, oral, and ocular regions are the other reported unusual localizations for MC.

MC may have atypical presentations including giant and eczematous lesions. The atypical manifestations may imitate many conditions including warts, basal cell carcinoma, intradermal nevus, and amelanotic melanoma [2]. The clinical diagnosis may be difficult, especially in HIV-infected patients. Other immunosuppressive conditions can also lead to atypical presentations. Our patient had no history of systemic disease and immunosuppression.

Dermoscopy has recently become an indispensable diagnostic tool in daily dermatology practice. Dermoscopic features of MC are well described. Roundish white-to-yellow structures and peripheral crown vessels are the typical dermoscopic findings. Radial and dotted vessels can also be seen. In our case, we observed a central yellowish structureless area surrounded by circumferential vessels. The main differential diagnosis was plantar verruca, in which dermoscopy usually reveals a lobulated surface with thrombosed capillaries giving a so-called “frogspawn appearance” in metaphorical language.

To the best of our knowledge, dermoscopic features of plantar MC have not been described previously.
